# Preparation of alginate coated chitosan microparticles for vaccine delivery

**DOI:** 10.1186/1472-6750-8-89

**Published:** 2008-11-19

**Authors:** XingYi Li, XiangYe Kong, Shuai Shi, XiuLing Zheng, Gang Guo, YuQuan Wei, ZhiYong Qian

**Affiliations:** 1 State Key Laboratory of Biotherapy, West China Hospital, West China Medical School, Sichuan University, Chengdu, 610041, PR China

## Abstract

**Background:**

Absorption of antigens onto chitosan microparticles via electrostatic interaction is a common and relatively mild process suitable for mucosal vaccine. In order to increase the stability of antigens and prevent an immediate desorption of antigens from chitosan carriers in gastrointestinal tract, coating onto BSA loaded chitosan microparticles with sodium alginate was performed by layer-by-layer technology to meet the requirement of mucosal vaccine.

**Results:**

The prepared alginate coated BSA loaded chitosan microparticles had loading efficiency (LE) of 60% and loading capacity (LC) of 6% with mean diameter of about 1 μm. When the weight ratio of alginate/chitosan microparticles was greater than 2, the stable system could be obtained. The rapid charge inversion of BSA loaded chitosan microparticles (from +27 mv to -27.8 mv) was observed during the coating procedure which indicated the presence of alginate layer on the chitosan microparticles surfaces. According to the results obtained by scanning electron microscopy (SEM), the core-shell structure of BSA loaded chitosan microparticles was observed. Meanwhile, *in vitro *release study indicated that the initial burst release of BSA from alginate coated chitosan microparticles was lower than that observed from uncoated chitosan microparticles (40% in 8 h vs. about 84% in 0.5 h). SDS-polyacrylamide gel electrophoresis (SDS-PAGE) assay showed that alginate coating onto chitosan microparticles could effectively protect the BSA from degradation or hydrolysis in acidic condition for at least 2 h. The structural integrity of alginate modified chitosan microparticles incubated in PBS for 24 h was investigated by FTIR.

**Conclusion:**

The prepared alginate coated chitosan microparticles, with mean diameter of about 1 μm, was suitable for oral mucosal vaccine. Moreover, alginate coating onto the surface of chitosan microparticles could modulate the release behavior of BSA from alginate coated chitosan microparticles and could effectively protect model protein (BSA) from degradation in acidic medium *in vitro *for at least 2 h. In all, the prepared alginate coated chitosan microparticles might be an effective vehicle for oral administration of antigens.

## Background

Development of an oral antigens (protein, and etc) delivery system for mucosal vaccine is a meaningful challenge for pharmaceutical scientists. The instability and poor absorption of antigens in gastrointestinal tract is major obstacles in the development of oral antigen delivery system for mucosal vaccine. Problems such as acid degradation in stomach, poor permeability across the gastrointestinal mucosa and the first-pass metabolism greatly limited the uptake of antigens by M-cell which is very important step for immune response [1,2]. To overcome the above-mentioned obstacles, several strategies, including liposomes [3-5], micro/nanoparticles [6-8], micro/nanoemulsion [9], and etc, have been explored to encapsulate antigens for the mucosal vaccine. Among these strategies, micro/nanoparticles made of biodegradable natural polymer have gained considerable interest in the past decades. One important aspect is that some natural polymers, especially chitosan, haves been demonstrated that could enhance the immunogenicity of poor immune response antigens in the form of solution and micro/nanoparticles [10,11].

Chitosan, as a cationic polysaccharide, has gained increasing attention in pharmaceutical field due to its favorable biological properties, such as non-toxicity, biodegradability [1,12], mucoadhesive properties [13,14], and etc. Additionally, chitosan micro/nanoparticles can be easily prepared by ionic gelation method using tripolyphosphate (TPP) as precipitating agent [12,15]. The advantage of this method was attributed to the mild condition without the application of harmful organic solvent at room temperature in the procedure, and also could efficiently detain the bioactivity of macromolecules (protein, DNA etc) during the encapsulation. In spite of all its superior properties, chitosan has an apparent pKa of 5.6 and is only soluble in acidic solutions. When incubated in physiological fluid environment, chitosan will lose its capacity of mucoadhesive properties and permeation enhancing effect due to the deprotonation of chitosan, which would make chitosan carriers lose its advantage compared with other carriers for mucosal vaccine. Meanwhile, chitosan has limited ability for controlling the release of encapsulated macromolecule compounds because of its hydrophilic nature and easy solubility in acidic medium [1,16]. It might be an interesting method to overcome these obstacles by coating acid-resistant polymer, such as alginate sodium, onto the surface of chitosan microparticles. As an anionic polysaccharide with favorable biological properties, alginate can easily interact with cationic chitosan microparticles to form the polyelectrolyte complex via electrostatic interactions [3,17-19]. Additionally, this coating procedure was performed at relatively mild condition without using any organic solvent. This relatively mild process has enabled not only proteins, but cells and DNA to be incorporated into the chitosan/alginate matrices with retention of biological activity [20].

In this work, we hope to develop a novel oral antigen carrier based on alginate coated chitosan microparticles to meet the requirement of mucosal vaccine. Model protein (BSA) was adopted to evaluate the properties of alginate coated chitosan microparticles. *In vitro *release behavior of BSA from chitosan microparticles and the stability of BSA loaded chitosan microparticles against acidic condition could be modified by alginate coating layer.

## Methods

### Materials

Chitosan with deacetylation (DA) of 92% and viscosity of 55 mpa.s (1% in 1% acetic acid, 20°C) was provided by Sigma (USA). Sodium alginate with viscosity of 20–40 cp (1% in distilled water) was obtained from Aldrich (USA). Bovine serum albumin (BSA) was purchased from BoAo Biochemical Company (Shanghai, China). Sodium tripolyphosphate was bought from Sigma (USA), and CaCl_2 _was bought from Chengdu KeLong Chemicals (Chengdu, China). BCA™ kit was provided by Pierce (USA). All other chemicals used in this paper were agent grade. Ultrapure water from Milli-Q water system was used to prepare the aqueous solutions.

### Preparation of chitosan microparticles

Chitosan microparticles were prepared by the ionic gelation of chitosan solution with anionic tripolyphosphate (TPP). Briefly, chitosan was dissolved in 1% (v/v) acetic acid aqueous solution at concentration of 5 mg/ml. Then, TPP was dissolved in distilled water at the concentration of 1 mg/ml. Subsequently, 9 ml of TPP solution was added dropwisely into 18 ml of chitosan solution (5 mg/ml), chitosan colloid microparticles were formed spontaneously under mild agitation at room temperature. Ten minutes later, chitosan colloid microparticles were centrifuged (Beckman Coulter™, Avanti™ J-30I centrifuge, Germany) at 9,500 rpm for 15 min. Then, the supernatant was discarded and the deposit was re-dispersed in distilled water for further use.

### Loading bovine serum albumin (BSA) to chitosan microparticles

Colloid chitosan microparticles were re-dispersed in 25 ml of distilled water at concentration of 5 mg/ml under continuous ultrasonication (Benchtop 20L, Medisafe, UK Ltd, UK) to disaggregate the chitosan microparticles. The loading procedure was performed by incubating different concentrations of BSA with chitosan microparticles under mild agitation at room temperature for 15 min. Loading efficiency (LE) and loading capacity (LC) of BSA on chitosan microparticles were detected in an indirect way by determining the free BSA remained in the supernatant after the performance of centrifuge, and the method was shown as following. One milliliter of BSA loaded chitosan microparticles suspension was centrifuged (Centrifuge 5415D, Eppendorf, Germany) at 13,200 rpm for 20 min and the amount of BSA in the supernatant was measured by BCA™ kit [8]. The supernatant of blank chitosan microparticles was adopted as the blank to correct the absorbance reading value of the BSA-loaded chitosan microparticles. The corrected optical density (OD) value was then used to calculate the concentration of BSA in the supernatant.

The loading efficiency (LE) and loading capacity (LC) values were calculated according to the following equations:

$$\begin{array}{c} \text{LE}(\%)=\frac{\text{total amount of BSA}-\text{free BSA}}{\text{total amount of BSA}}\times 100\\ \text{LC}(\%)=\frac{\text{total amount of BSA}-\text{free BSA}}{\text{dried microparticle weight}}\times 100 \end{array}$$

### Preparation of alginate coated chitosan microparticles

BSA loaded chitosan microparticles suspensions with pH value at 5.1 were added dropwisely into sodium alginate solution (pH = 7.2) at concentration of 10 mg/ml under mild agitation for 10 min. Then the suspension was centrifuged at 3,400 rpm for 5 min, and the supernatant was discarded. Finally, alginate coated chitosan microparticles were re-dispersed into calcium chloride (CaCl_2_) aqueous solution (pH = 7.0) at concentration of 0.524 mmol/L to crosslink the alginate layer presents on the surface of chitosan microparticles.

### Morphological characterization, size and surface charge

The morphological characteristics of microparticles were examined by scanning electron microscopy (JSM-5900LV, JEOL, Japan). Microparticles were sputtered with gold and maintained at room temperature for complete dryness before the observation.

The particle size distribution was detected by laser diffraction (Nano-ZS 90, Malvern Instrument, UK; BT-2002 Laser Particle Size Analyzer, Dandong Bettersize Instruments LTD, China). The zeta potential of particles was examined by Malvern Zeta analyzer (Nano-ZS 90, Malvern Instrument, UK) with ultrapure water as solvent (pH = 7, 25°C). These measurements were run at least three times with independent particle batches.

### Protein release *in vitro*

*In vitro *release behavior of BSA from uncoated and alginate coated chitosan microparticles were determined as followed. One milliliter of microparticles suspension was first centrifuged and the deposit was incubated in 1 ml of phosphate buffer saline (PBS, pH7.4) in Eppendorf tube (EP tube). Then, the EP tube was placed in an air shaker bath at 100 rpm/min (at 37°C) for *in vitro *release. At scheduled time, samples were centrifuged at 13,200 rpm for 20 min and the supernatant was replaced with fresh PBS (pre-warmed to 37°C). The amount of BSA presented in the supernatant was determined by BCA™ kit as described in **section 2.3**. According to protocol, the amount of BSA released was expressed as a percentage of total BSA encapsulated in chitosan microparticles as calculated from the LE value.*In vitro *release experiments were repeated three times.

### Acidic degradation protection assay

Different formulations of chitosan microparticles (BSA loaded chitosan microparticles and alginate coated BSA loaded chitosan microparticles) were first centrifuged at 13,000 rpm for 20 min and the supernatant was discarded. Then, the deposition was incubated with 0.5 ml of HCl (0.01 M) in air shaker bath at 37°C for 2 h. Finally, the reaction was stopped by 0.5 ml of aqueous NaOH (0.01 M) solution. These systems were sustained release for another 24 h with addition of PBS to final volume at 4 ml. Twenty four hours later, the supernatant containing released BSA was collected and analyzed by SDS-polyacrylamide gel electrophoresis (SDS-PAGE). The pure BSA solution was designed as the control.

### Fourier transform infra-red (FTIR) measurements

FTIR measurements were taken at room temperature using NICOLET 200SXV Infrared Spectrophotometer (USA). Alginate coated chitosan microparticles at 0 h and 24 h of release test in PBS (pH = 7.4) were centrifuged and washed with ultrapure water, finally freeze-dried overnight before the detection.

## Results and discussion

### Preparation of alginate coated BSA loaded chitosan microparticles

In this article, we prepared alginate coated chitosan microparticles by layer-by-layer technology to meet the requirement of oral administration of antigen for mucosal vaccine. BSA with isoelectric point (PI) of 4.8 was negatively charged when pH>4.8, which could easily absorb cationic chitosan microparticle at aqueous solution (pH = 7) via electrostatic interaction and was selected as the model protein to evaluate the properties of alginate coated chitosan microparticles. This procedure could be divided into three parts, preparation of the chitosan microparticles, loading of BSA onto chitosan microparticles, and finally coating of BSA loaded chitosan microparticles with sodium alginate. Chitosan microparticles were prepared by ionic gelation method using sodium tripolyphosphate (TPP) as precipitating agent that has been described by several authors [12,15]. According to Table 1, the mean particle size of prepared chitosan microparticles was about 300 nm and the PDI was 0.309. Then, chitosan microparticles were incubated in aqueous solution with different BSA concentrations at a relatively mild condition to obtain suitable loading efficiency (LE) and loading capacity (LC). Finally, alginate was introduced to coat onto surface of BSA loaded chitosan microparticles to increase the acidic resistance of BSA loaded chitosan microparticles and to modulate the release behavior of BSA from chitosan microparticles [4,17]. Effect of alginate/chitosan microparticles weight ratio on the properties of microparticles was presented in Table 2. As shown in Table 2, we could find that stable alginate coated BSA loaded chitosan microparticles could be obtained when the weight ratio of alginate/chitosan microparticles was greater than 2. It might be explained by the fact that the addition of small amount of alginate (the weight ratio of alginate/chitosan microparticles smaller than 2) to chitosan microparticles decreased the zeta potential of microparticles and could facilitate the gelation of chitosan and alginate resulting in agglomeration or precipitation [21,22]. Meanwhile, when the weight ratio of alginate/chitosan microparticles was greater than 2, the rapid charge inversion and gelation lead to the formation of stable chitosan microparticles. Calcium ion (CaCl_2_, 0.524 mM) was used as crosslinking agent to strengthen and stabilize the particles [22].

**Table 1:** The size and zeta potential of alginate/BSA/chitosan system

Sample	Mean particles size (nm)	PDI	Zeta potential (mV)
Blank chitosan microparticles ^a^	301.8	0.309	+45.2
BSA-loaded chitosan particles ^b^	404	0.472	+27.1
Alginate coated BSA loaded chitosan microparticles ^c^	1324	0.450	-27.8

^a ^chitosan 5 mg/ml, TPP 1 mg/ml.

^b ^chitosan 5 mg/ml, TPP 1 mg/ml, BSA 2 mg/ml.

^c ^chitosan 5 mg/ml, TPP 1 mg/ml, BSA 2 mg/ml, alginate 10 mg/ml.

**Table 2:** Effect of alginate/chitosan microparticles weight ratio on the properties of alginate -chitosan microparticles formulation

Alginate/chitosan microparticles weight ratio	Mean particles size (nm)
1:1	Immediately precipitation
2:1	2722 (partially precipitation)
3:1	1324
4:1	1021

### Procedure of protein loading

#### Effect of BSA concentration

**Figure 1:**
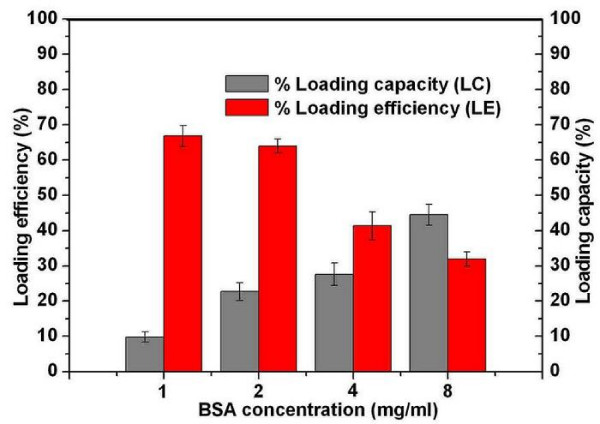
**Effect of different BSA concentrations on the loading efficiency (LE) and loading capacity (LC) of chitosan microparticles**.

Cationic microparticles can easily absorb anionic protein or DNA via electrostatic interaction [23,24]. Cationic chitosan microparticles prepared in this work had a potent capacity to absorb the model anionic protein (BSA) in aqueous solution via electrostatic interaction. The obtained chitosan microparticles at concentration of 5 mg/ml were adopted to evaluate particle size, zeta potential, loading efficiency, and loading capacity of alginate coated BSA loaded chitosan microparticles. As presented in Table 3, effect of BSA concentration on the properties of chitosan microparticle were investigated. The mean diameter of chitosan microparticles increased accompanied with decrease in zeta potential when BSA concentration increased from 1 mg/ml to 8 mg/ml. This might be attributed to the fact that negatively charged BSA absorbed onto chitosan microparticles and neutralized part of zeta potential of chitosan microparticles resulted in increase of particle size and decrease of zeta potential [25]. Fig. 1 depicts the effect of BSA concentration on the loading efficiency (LE) and loading capacity (LC) of chitosan microparticles. When the concentration of BSA was lower than 2 mg/ml, the loading efficiency was greater than 60%. The loading efficiency significantly decreased to about 30 % while BSA concentration was greater than 2 mg/ml. This interesting phenomenon might be attributed to the saturated absorption was achieved as BSA concentration at about 2 mg/ml, the more addition of BSA was seldom adsorbed onto chitosan microparticles which leaded to the great decrease in loading efficiency (LE). Meanwhile, loading capacity increased from 10% to 40% as the BSA concentration increased from 1 mg/ml to 8 mg/ml.

**Table 3:** The mean particles size and zeta potential of BSA loaded chitosan microparticles

Sample	BSA concentration (mg/ml)	Mean particle size (nm)	PDI	Zeta potential (mV)
Chitosan microparticles at concentration of 5 mg/ml	1	380.8	0.393	+34.0
	2	404.0	0.472	+27.1
	4	420.3	0.450	+22.5
	8	464.1	0.499	+12.7

#### Effect of alginate/chitosan microparticles weight ratio

**Figure 2:**
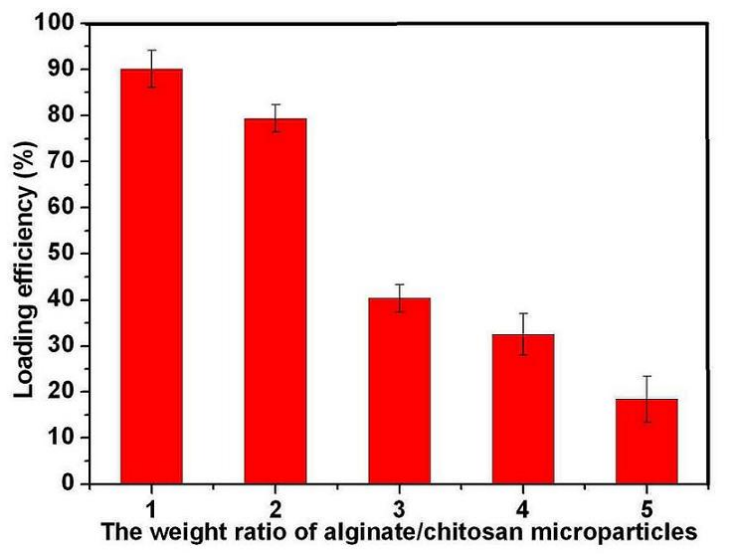
**Effect of alginate/chitosan microparticles weight ratio on loading efficiency (LE) of alginate coated chitosan microparticles**.

Due to the electrostatic interaction between the positively charged -NH_3_^+ ^of chitosan microparticles and negatively charged -COO^- ^of alginate, cationic BSA-loaded chitosan microparticles could be easily modified with anionic alginate in aqueous solution via electrostatic interaction [26]. However, the addition of anionic alginate to cationic BSA loaded chitosan microparticles would replace some of BSA absorption on the chitosan microparticles due to the competitive electronic interaction [21]. According to Fig. 2, we could find that the loading efficiency (LE) of BSA on chitosan microparticles increased with decrease in alginate/chitosan microparticles weight ratio until the mass ratio of alginate/chitosan microparticles was smaller than 2, but the precipitation of chitosan microparticles was also observed. The zeta potential of colloidal chitosan microparticles is an important factor for the stability of particles. Generally, the higher zeta potential of particles led to more stability of colloidal particles, and the lower zeta potential of particles induced agglomeration of colloidal particles [12,21]. The observed precipitation of system was probably caused by the decrease in zeta potential (from +27 mv to -0.4 mv) and the gelation of chitosan with addition of alginate. As the precipitation proceeded, the higher LE was observed which might be attributed to co-precipitation of BSA because of the gelation of chitosan and alginate. However, stable colloidal microparticle system (no precipitation observed) could be obtained when the weight ratio of alginate/chitosan microparticles was greater than 2, which might be explained by that the rapid charge inversion and coating process of microparticles (from +27 mv to -27.8 mv) leading to the stable system formed. However, the loading efficiency (LE) and loading capacity (LC) of BSA on chitosan microparticles decreased due to the competitive adsorption between alginate and BSA resulting in some BSA desorption from chitosan microparticles [21,27].

#### Characterization of chitosan microparticles

**Figure 3:**
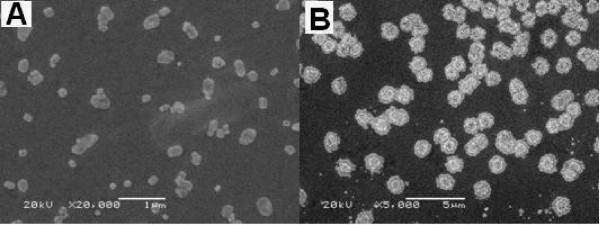
**SEM images of chitosan microparticles (A) and alginate coated BSA loaded chitosan microparticles (B)**.

**Figure 4:**
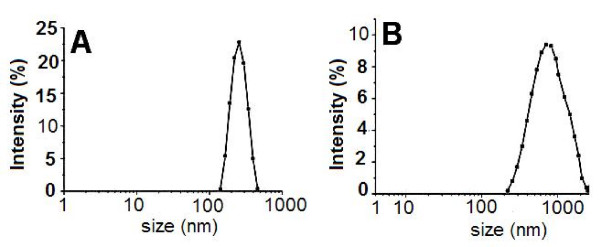
**The particle size distribution of chitosan microparticles (A), and alginate coated BSA loaded chitosan microparticles (B)**.

The obtained chitosan microparticles and alginate coated BSA loaded chitosan microparticles were characterized by SEM and laser diffraction. Two typical scanning electron microphotographs of chitosan microparticles and alginate coated BSA loaded chitosan microparticles were presented in Fig. 3(A–B), respectively. According to Fig. 3(A), chitosan microparticles were almost irregular spherical with size ranging from 50 to 300 nm. It was also observed that some small chitosan microparticles fused into large ones. The result detected by Malvern Instrument indicated that the average size of chitosan microparticles was about 300 nm and zeta potential was +45.2 mv (Table 2). The size distribution of chitosan microparticles was presented in Fig. 4(A), which indicated that well dispersed chitosan microparticles were prepared. As shown in Fig. 3(B), SEM photograph of alginate coated BSA loaded chitosan microparticles show that the mean size of microparticles was about 1 μm which was larger than BSA loaded chitosan microparticles due to the coating microparticles with alginate and the morphology of alginate coated BSA loaded chitosan microparticles was shown to have core-shell structure which indicated that the sodium alginate was successfully coated on the surface of BSA loaded chitosan microparticles core. Here, we also found that some particles were adhered together. The size distribution of alginate coated BSA loaded chitosan microparticles was presented in Fig. 4(B). During the coating procedure, the inversion of the zeta potential was observed by the Malvern Instrument. After complete coating of alginate on chitosan microparticles core, the zeta potential of chitosan microparticles was about -27.8 mv (Table 1), which also implied the presence of alginate layer on the surface of chitosan microparticles.

#### Protein release *in vitro*

**Figure 5:**
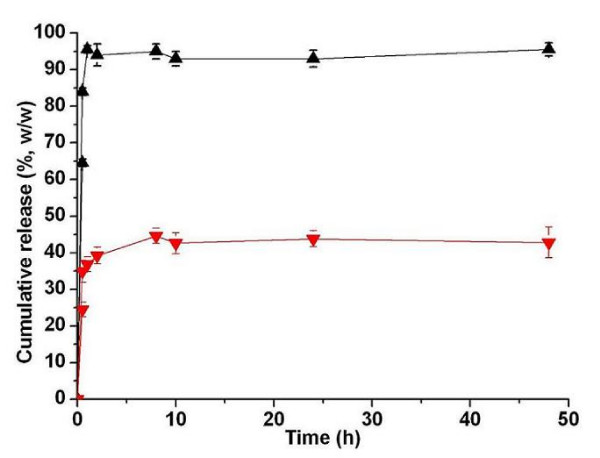
***In vitro*****release profiles of BSA from uncoated chitosan microparticles (Black) and alginate coated chitosan microparticles (Red) in PBS (pH7.4) at 37°C**.

The release profiles of BSA from uncoated and alginate coated chitosan microparticles at phosphate buffer (PBS, pH7.4) were shown in Fig. 5. As depicted in Fig. 5, the initial burst release (about 84%) of BSA from uncoated chitosan microparticles occurred in the first 0.5 h, followed by release of 95.5% in 2 days. The burst release might be attributed to the fact that BSA macromolecules were loosely bound onto chitosan microparticles by ionic interaction which could be easily desorbed at ionic environment [27]. However, alginate modified chitosan microparticle could increase the stability of chitosan microparticles in the PBS at 37°C which resulted in extended release of BSA, only about 40% of BSA released in 8 h. And 48 h later, there was still about 50% of BSA retained in the alginate coated chitosan microparticles. The total ratio of BSA released from the alginate coated chitosan microparticles in 48 h was much less than that observed from uncoated chitosan microparticles. The longer release time and slower release rate of BSA from alginate coated chitosan microparticles might be explained by that there are strong interaction between polymers (chitosan and alginate) and BSA [28]. Additionally, the presence of alginate layer could slow down the diffusion of BSA from the system which also prolong the release time of BSA from system. But, due to both the degradation and erosion of matrix, the BSA might be released from the alginate/BSA/chitosan formulation in an extended profile [29]. This release behavior studies indicated that coating of alginate onto chitosan microparticles could improve the stability of chitosan microparticles in PBS and modify the release behavior of BSA from these alginate coated chitosan microparticles.

#### Acidic degradation protection

**Figure 6:**
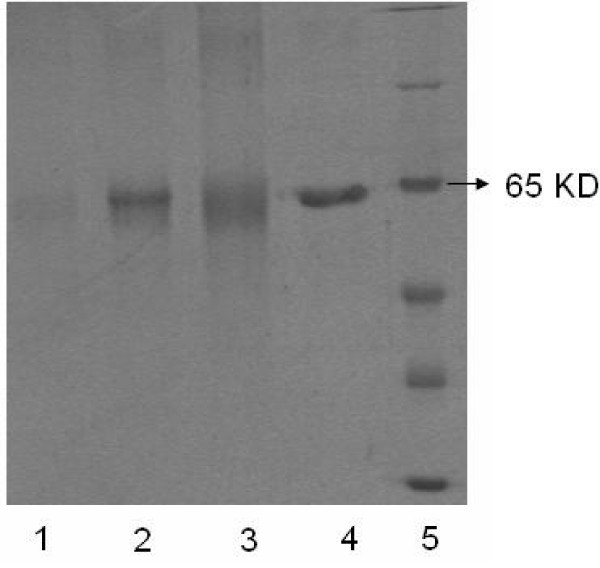
**SDS-PAGE patterns, obtained under conditions of uncoated chitosan microparticles containing BSA pretreated with 0.01 M HCl for 2 h then sustained release in PBS for 24 h (Lane 1), alginate coated chitosan microparticles containing BSA pretreated with 0.01 M HCl for 2 h then sustained release in PBS for 24 h (Lane 2), BSA pretreated with 0.01 M HCl for 2 h then incubation with PBS for another 24 h (Lane 3), BSA incubation with PBS for 24 h (Lane 4), Molecular weight marker (Lane 5)**.

In this paper, we hope to design an oral antigens delivery system for mucosal vaccine. As we know, pH value of gastric fluid is approximately 2 which will destroy the integrity and structure of antigens (protein, DNA, etc) after the oral administration without any protection. Therefore, it is very important for us to develop an antigen carrier based on chitosan and alginate that can effectively avoid the acidic degradation of encapsulated antigen before it reached the target site. Here, BSA released from uncoated and alginate coated chitosan microparticles under acidic medium (pH2.0) and PBS medium (pH7.4) was analyzed by SDS-polyacrylamide gel electrophoresis (SDS-PAGE). According to Fig. 6, molecular weight marker was shown in Lane 5, and the BSA incubated with PBS (pH7.4) for 24 h exhibited a clear band at about 66 KD (Lane 4). However, BSA pretreated with 0.01 M HCl for 2 h then incubated with PBS for 24 h had a faint band at 66 KD (Lane 3) which indicated the serious degradation or hydrolysis of BSA in acidic medium. The SDS-PAGE gel banding patterns of BSA released from uncoated and alginate coated chitosan microparticles with HCl (pH2.0) pretreatment for 2 h and then sustained release in PBS for 24 h are shown in Lane 1 and Lane 2, respectively. It is seen that BSA from uncoated chitosan microparticles had a very weak band at 66 KD (Lane 1) which indicated that the BSA underwent hydrolysis/degradation in spite of presence of chitosan microparticles (pH2.0). Meanwhile, we also observed the flocculent precipitate of system with the addition of sodium hydrate to BSA loaded chitosan microparticles which implied the dissolution of chitosan microparticles in acidic condition (pH2). However, BSA from alginate coated chitosan microparticles had a clear band at about 66 KD (Lane 2), which implied that alginate coating of chitosan microparticles could effectively protect BSA from hydrolysis/degradation at acidic medium for at least 2 h. So, the obtained alginate coated chitosan microparticles might be an effective oral antigenic carrier for mucosal vaccine

#### Fourier transform infra-red (FTIR) measurements

**Figure 7:**
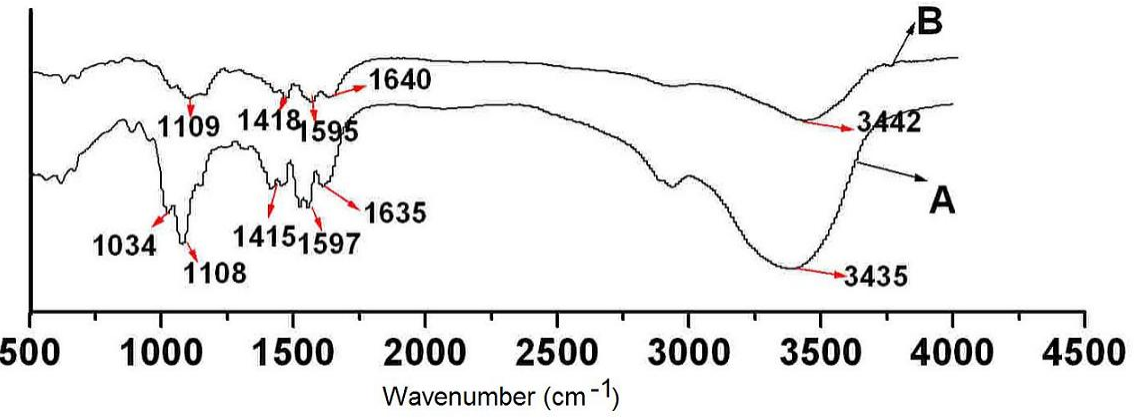
**FTIR spectra of alginate coated chitosan microparticles at 0 h (A) and 24 h (B) of release test using pH7.4 phosphate buffer**.

Stability of alginate coated chitosan microparticles in PBS was investigated using FTIR spectroscopy. According to Fig. 7, alginate coated chitosan microparticles at 0 h and 24 h of release test showed similar spectra. The peaks at 3435, 1640, 1415 and 1109 cm^-1 ^were due to the stretching of O-H, COO^-^(asymmetric), COO^-^(symmetric), and C-O-C, respectively. Compared with FTIR spectrum of alginate modified chitosan microparticles at 0 h of release test, the shift of COO^- ^peaks at 1635 and 1415 cm^-1 ^to higher wavenumber (1640 and 1418 cm^-1 ^respectively), as well as the decrease in intensity, was observed in alginate modified chitosan microparticles at 24 h of release test in PBS, which indicated that ionic desorption between COO^- ^of alginate and calcium ions or NH_3_^+ ^of chitosan was occurred at 24 h of release test [30,31].

## Conclusion

The prepared alginate coated chitosan microparticles, with mean diameter of about 1 μm, was suitable for oral administration. Moreover, alginate coating onto surface of chitosan microparticles could modulate the release behavior of BSA from alginate coated chitosan microparticles and could effectively protect model protein (BSA) from degradation against acidic medium (pH2) *in vitro *at least for 2 hours. According to FTIR, some alginate on surface of chitosan microparticles at 24 h of release test has been dissolved into PBS. Based on the information demonstrated, the prepared alginate coated chitosan microparticles might be an effective vehicle for oral administration of antigens.

## Abbreviations

BSA: bovine serum albumin; LE: loading efficiency; SEM: scanning electron microscopy; SDS-PAGE: SDS-polyacrylamide gel electrophoresis; FTIR: Fourier transform infra-red; TPP: tripolyphosphate; PI: isoelectric point; LC: loading capacity; DA: deacetylation; OD: optical density; EP: Eppendorf tube; HCl: hydrochloric acid; CaCl_2_: calcium chloride; NaOH: sodium hydroxide.

## Competing interests

The authors declare that they have no competing interests.

## Authors' contributions

QZY, WYQ, LXY, and KXY designed the experiments. And the research funds were supported by QZY and WYQ. LXY and KXY carried out experiments, analyzed the data, and wrote the manuscript, and KXY is the co-first author for this paper; QZY and GG corrected the manuscript. SS did the SDS-PAGE experiment. ZXL studied the in vitro release behavior of the microparticles.

All authors approved and read the final manuscript.

## References

[B1] George M, Abraham TE (2006). Polyionic hydrocolloids for the intestinal delivery of protein drugs: alginate and chitosan -a review. J Control Release.

[B2] Jepson MA, Clark MA, Hirst BH (2004). M cell targeting by lectins: a strategy for mucosal vaccination and drug delivery. Adv Drug Deliv Rev.

[B3] Okada E, Sasaki S, Ishii N, Aoki I, Yasuda T, Nishioka K, Fukushima J, Miyazaki J, Wahren B, Okuda K (1997). Intranasal immunization of a DNA vaccine with IL-12-and granulocyte-macrophage colony-stimulating factor (GM-CSF)-expressing plasmids in liposomes induces strong mucosal and cell-mediated immune responses against HIV-1 antigens. J Immunol.

[B4] Wang W (1996). Oral Protein Drug Delivery. J Drug Target.

[B5] Anderson KE, Eliot LA, Stevenson BR, Rogers JA (2001). Formulation and Evaluation of a Folic Acid Receptor-Targeted Oral Vancomycin Liposomal Dosage Form. Pharm Res.

[B6] Vila A, Sánchez A, Tobío M, Calvo P, Alonso MJ (2002). Design of biodegradable particles for protein delivery. J Control Release.

[B7] Lubben MVD, Kersten G, Fretz MM, Beuvery C, Verhoef JC, Junginger HE (2003). Chitosan microparticles for mucosal vaccination against diphtheria: oral and nasal efficacy studies in mice. Vaccine.

[B8] Lubben MVD, van Opdorp FAC, Hengeveld MR, Onderwater JJM, Koerten HK, Verhoef JC, Borchard G, Junginger HE (2002). Transport of Chitosan Microparticles for Mucosal Vaccine Delivery in a Human Intestinal M-cell Model. J Drug Target.

[B9] Bielinska AU, Janczak KW, Landers JJ, Makidon P, Sower LE, Peterson JW, Baker JR (2007). Mucosal Immunization with a Novel Nanoemulsion-Based Recombinant Anthrax Protective Antigen Vaccine Protects against Bacillus anthracis Spore Challenge. Infect Immun.

[B10] Zaharoff DA, Rogers CJ, Hance KW, Schlom J, Greiner JW (2007). Chitosan solution enhances both humoral and cell-mediated immune responses to subcutaneous vaccination. Vaccine.

[B11] Amidi M, Romeijn SG, Coos Verhoef J, Junginger HE, Bungener L, Huckriede A, Crommelin DJA, Jiskoot W (2007). N-Trimethyl chitosan (TMC) nanoparticles loaded with influenza subunit antigen for intranasal vaccination: Biological properties and immunogenicity in a mouse model. Vaccine.

[B12] Gan Q, Wang T, Cochrane C, McCarron P (2005). Modulation of surface charge, particle size and morphological properties of chitosan-TPP nanoparticles intended for gene delivery. Colloids Surfaces B.

[B13] He P, Davis SS, Illum L (1998). In vitro evaluation of the mucoadhesive properties of chitosan microspheres. Int J Pharm.

[B14] Schnurch AB, Humenberger C, Valenta C (1998). Basic studies on bioadhesive delivery systems for peptide and protein drugs. Int J Pharm.

[B15] Berthold A, Cremer K, Kreuter J (1996). Preparation and characterization of chitosan microsphere as drug carrier for prednisolone sodium phosphate as model for anti-inflammatory drugs. J Control Release.

[B16] Kotzé AF, Lueßen HL, de Boer AG, Verhoef JC, Junginger HE (1999). Chitosan for enhanced intestinal permeability: prospects for derivatives soluble in neutral and basic environments. Eur J Pharm Sci.

[B17] Lee BJ, Min GH (1996). Oral controlled release of melatonin using polymer- reinforced and coated alginate beads. Int J Pharm.

[B18] Kim B, Bowersock T, Griebel P, Kidane A, Babiuk LA, Sanchez M, Attah-Poku S, Kaushik RS, Mutwiria GK (2002). Mucosal immune responses following oral immunization with rotavirus antigens encapsulated in alginate microspheres. J Control Release.

[B19] Severian D, Esteban C (1998). Inclusion and release of proteins from polysaccharide-based polyion complexes. Adv Drug Deliv Rev.

[B20] Gombotz WR, Wee SF (1998). Protein release from alginate matrices. Adv Drug Deliv Rev.

[B21] Borges O, Borchard G, Verhoef JC, de Sousa A, Junginger HE (2005). Preparation of coated nanoparticles for a new mucosal vaccine delivery system. Int J Pharm.

[B22] Borges O, Cordeiro-da-Silva A, Romeijn SG, Amidi M, Sousa AD, Borchard G, Junginger HE (2006). Uptake studies in rat peyer's patches, cytotoxicity and release studies of alginate coated chitosan nanoparticles for mucosal vaccination. J Control Release.

[B23] Illum L, Jabbal-Gill I, Hinchcliffe M, Fisher AN, Davis SS (2001). Chitosan as a novel nasal delivery system for vaccines. Adv Drug Deliv Rev.

[B24] Cui Z, Mumper RJ (2001). Chitosan-based nanoparticles for topical genetic immunization. J Control Release.

[B25] Xu YM, Du YM (2003). Effect of molecular structure of chitosan on protein delivery properties of chitosan nanoparticles. Int J Pharm.

[B26] Fan YF, Wang YN, Fan YG, Ma JB (2006). Preparation of insulin nanoparticles and their encapsulation with biodegradable polyelectrolytes via the layer-by-layer adsorption. Int J Pharm.

[B27] Chen F, Zhang ZR, Huang Y (2007). Evaluation and modification of N-trimethyl chitosan chloride nanoparticles as protein carriers. Int J Pharm.

[B28] Coppi G, Iannuccelli V, Leo E, Bernabei MT, Cameroni R (2001). Chitosan-Alginate Microparticles as a Protein Carrier. Drug Dev Ind Pharm.

[B29] Anal AK, Bhopatkar D, Tokura S, Tamura H, Stevens WF (2003). Chitosan-Alginate Multilayer Beads for Gastric Passage and Controlled Intestinal Release of Protein. Drug Dev Ind Pharm.

[B30] Pongjanyakul T, Puttipipatkhachorn S (2007). Modulating drug release and matrix erosion of alginate matrix capsules by microenvironmentral interaction with calcium ion. Eur J Pharm Biopharm.

[B31] Puttipipatkhachorn S, Pongjanyakul T, Priprem A (2005). Molecular interaction in alginate beads renforced with sodium starch glycolate or magnesium aluminum silicate, and their physical characteristics. Int J Pharm.

